# *K*-Volume Clustering Algorithms for scRNA-Seq Data Analysis

**DOI:** 10.3390/biology14030283

**Published:** 2025-03-11

**Authors:** Yong Chen, Fei Li

**Affiliations:** 1 Department of Biological and Biomedical Sciences, Rowan University, Glassboro, NJ 08028, USA; chenyong@rowan.edu; 2 Department of Computer Science, George Mason University, Fairfax, VA 22030, USA

**Keywords:** single-cell omics, clustering algorithms, gene regulatory networks

## Abstract

Clustering high-dimensional and structural data remains a key challenge in computational biology, especially for complex single-cell and multi-omics datasets. In this study, we present *K*-volume clustering, a novel algorithm that uses the total convex volume defined by points within a cluster as a biologically relevant and geometrically interpretable criterion. This method simultaneously optimizes both the hierarchical structure and the number of clusters at each level through nonlinear optimization. Validation on real datasets shows that *K*-volume clustering outperforms traditional methods across a range of biological applications. With its theoretical foundation and broad applicability, *K*-volume clustering holds great promise as a core tool for diverse data analysis tasks.

## Simple Summary

Clustering high-dimensional and structural data remains a significant challenge in computational biology, particularly for complex single-cell and multi-omics datasets. In this work, we introduce a novel clustering algorithm that utilizes the total convex volume defined by points within a cluster as a biologically relevant and geometrically interpretable criterion. This approach simultaneously optimizes both the hierarchical structure and the number of clusters at each level through nonlinear optimization. We evaluate our algorithm against other clustering methods, and the results demonstrate that our approach outperforms traditional techniques across a variety of biological applications.

## 1. Introduction

Clustering is a fundamental task in data analysis, aiming to group similar data points based on their intrinsic properties and patterns, without relying on labeled data [1]. Among the various clustering methods, *K*-means and its variants are widely recognized for their simplicity, scalability, and efficiency [2,3]. The *K*-means algorithm partitions a dataset into *K* clusters by iteratively minimizing intra-cluster variance and maximizing inter-cluster separation. Variants such as *K*-center [4], *K*-median [5] and *K*-density [6] have been introduced to better handle diverse data distributions, improve robustness to outliers, and capture nonlinear relationships. However, these methods typically require the number of clusters, *K*, to be predefined, which presents a significant challenge, especially with complex datasets.

Clustering methods have become essential tools in biological data analysis, particularly with the rise of high-throughput, high-dimensional datasets like single-cell RNA sequencing (scRNA-seq) [7]. scRNA-seq provides a powerful means of analyzing gene expression at the single-cell level, enabling the identification of diverse cell types, states, and lineages. In this context, clustering is critical for grouping cells with similar expression profiles to uncover cell heterogeneity and infer biological function [8,9]. Methods such as *K*-means, hierarchical clustering, and graph-based clustering have been adapted to handle the high dimensionality and sparsity of scRNA-seq data [7,10]. Advanced variations, including those that integrate dimensionality reduction techniques like Principal Component Analysis (PCA) or t-SNE, have further enhanced the interpretability of scRNA-seq clusters, facilitating the discovery of novel cell populations and their functional characteristics [11].

Identifying the optimal number of clusters (*K*) and defining hierarchical layers (*H*) remain significant challenges in both theoretical analysis and practical applications [3]. In *K*-means clustering, the requirement to predefine *K* can lead to complications; improper choices may result in over- or under-clustering, distorting the underlying data structure. Similarly, hierarchical clustering lacks a universal criterion for determining the appropriate number of layers or meaningful cut points in the dendrogram, often leading to subjective or inconsistent interpretations [12]. Techniques like the elbow method [13], silhouette analysis [14], and gap statistics [15] have been developed to estimate *K*, but these methods are frequently computationally expensive and highly sensitive to dataset characteristics. This issue is particularly pronounced in single-cell RNA sequencing (scRNA-seq) data, where biological processes span multiple scales and resolutions [16]. Additionally, the number of clusters can vary depending on the specific objectives, such as identifying common cell populations or detecting rare cell types [7,9]. The hierarchical relationships between cell populations, shaped by lineage progression and differentiation pathways, further complicate the analysis [17,18]. Therefore, there is a critical need for robust, automated approaches that can simultaneously determine the number of clusters and uncover hierarchical structures, especially given the complex, high-dimensional nature of scRNA-seq data.

**Figure 1:**
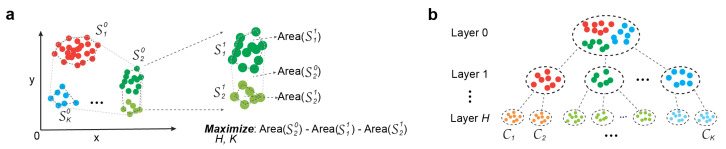
Illustration of optimization strategies: (**a**) Convex hull-based optimization. (**b**) Identification of cell types and their hierarchical organization from scRNA-seq data.

In this study, we introduce *K*-volume clustering, a novel and foundational algorithm for optimal hierarchical clustering. This algorithm simultaneously optimizes both the number of hierarchical layers *H* and the number of clusters *K* within each layer using nonlinear optimization principles (see Figure 1). At each level, it maximizes the difference between the area of the convex hull encompassing all sample points and the cumulative areas of the *K* sub-convex hulls (Figure 1a). By jointly optimizing *H* and *K*, the method determines the optimal hierarchical structure, resulting in a robust clustering solution (Figure 1b). This new approach is particularly useful for revealing novel insights from complex biological datasets, enabling a deeper understanding of biological architectures and potentially uncovering hidden functional or evolutionary relationships.

### 1.1. Related Work

Clustering partitions data points into subsets, where each subset forms a cluster based on a specified criterion [1]. Clustering algorithms group ‘similar’ data points to uncover the relationships among them. Common similarity measures include Euclidean distance [19], squared Euclidean distance [20,21], and Hamming distance [22].

Clustering algorithms are generally categorized into center-based and density-based methods. Specifically, the *K*-volume clustering algorithm defines the “center” as an inner point of the convex hull formed by all the points in the cluster, which aligns it with the characteristics of a center-based clustering method. At the same time, the size of the convex hull defined by the *K*-volume algorithm corresponds to the “density” of the cluster. Thus, our *K*-volume clustering method effectively integrates aspects of both approaches.

Table 1 summarizes widely used clustering algorithms, with entries marked by ∗ denoting our contributions in this paper. Notably, center-based clustering problems are NP-hard, and finding optimal *K*-means clusters remains NP-hard, even in two-dimensional space [23]. Table 1 also lists the most recent approximation algorithms for these clustering problems.

In the work of Crescenzi and Giuliani [26], the authors identified mutual similarities from cluster analysis among differently labeled statistical units, generating new classifications at varying levels of detail that can be interpreted in biological terms. Similar to this work, our work offers another effort in the clustering analysis of complex scRNA-seq data at multiple levels of detail.

### 1.2. Our Contribution and Paper Organization

In this paper, we develop algorithms specifically for clustering structural data, such as biological and single-cell omics. In particular, we propose a novel clustering algorithm that uses the total convex volume enclosed by points within the same cluster as a clustering measure.

We present an intuitive and efficient algorithm along with its theoretical analysis in Section 2. In Section 3, we evaluate its performance against well-established clustering algorithms on biological data. Finally, we conclude our paper in Section 5.

## 2. A Greedy *K*-Volume Clustering Algorithm

We consider a clustering problem with the following input: a set of *N* data points, $p_{1},p_{2},\ldots ,p_{N}$, where each point is represented as a *D*-dimensional real vector in $R^{D}$. The distance between any two points, $p_{i}$ and $p_{j}$, is denoted by $d(p_{i},p_{j})$. We introduce a new measure based on the convex volume. Specifically, the clustering cost is defined as the minimal convex volume enclosing all points within a cluster. Under this definition, a single data point has a volume of 0, and any set of collinear points also has a volume of 0.

We define the problem of partitioning data into *K* clusters within a hierarchical structure while minimizing the total convex volume as the *K*-volume clustering problem. When the dimensionality is D=2, we refer to it as the *K*-area clustering problem.

### 2.1. The Algorithm’s Idea

We propose a simple yet elegant algorithm based on a greedy approach. In this work, we illustrate the idea for the case where D=2, with the approach being naturally extendable to higher dimensions (D≥3). The algorithm starts with the convex hull of the data points. The key idea is to partition a cluster into two in such a way that the convex volume of the remaining clusters is minimized. This process is repeated iteratively until the total number of clusters reaches the specified value *K* or the given maximal hierarchical level *H*.

Given a set of points, finding the initial convex hull takes time $O(n^{\lfloor D/2\rfloor +1})$ [27]. The main algorithmic challenges in finding *K*-volume clusters are (1) how to partition the data points into *K* clusters, and (2) how to calculate the total volume of these clusters.

Consider a set of *N* points, $p_{1},p_{2},\ldots ,p_{N}$, where each point $p_{i}$ is described by its coordinates $\left(x_{i},y_{i}\right)$ in 2D space. Without loss of generality, we assume all *x*-axis and *y*-axis values are non-negative. Our goal is to partition the points into *K* clusters, i.e., *K* convex areas on the 2D plane, such that the total area is minimized. Let *S* represent a set of points, and A(S) denote the area of the convex polygon enclosing *S*.

The algorithm proceeds as follows: Initially, using Graham’s scan [28], we calculate the convex polygon for all *N* points. If K=1, this convex polygon is the optimal solution, and all points belong to the same cluster. If K≥2, we proceed to build a tree that represents the set of clusters created during the execution of the algorithm. The tree structure allows the algorithm to efficiently organize and partition the data into meaningful groups. Since each layer in the tree represents a finer partitioning, the algorithm benefits from the hierarchical structure when it comes to refining clusters, especially as the number of clusters increases. The tree has the following properties:

1. The root node represents the initial convex polygon for all the data points.
2. Each node in the tree corresponds to a cluster. The root of each subtree represents the cluster for all the data points clustered in its subtree.
3. The leaf nodes represent the set of clusters that we currently have at any point in time.

For K≥2, we iteratively partition one of the current clusters, say *C*, into two subclusters, $C^{1}$ and $C^{2}$. These subclusters, $C^{1}$ and $C^{2}$, become the children of node *C*. This partitioning process continues until we have exactly *K* clusters or reach the maximal hierarchical level *H*. When partitioning a cluster *C* into two subclusters $C^{1}$ and $C^{2}$, we use a greedy approach that maximizes the size of the removed area, ensuring an optimal division.

$$A\left(C\right)-[A\left(C^{1}\right)+A\left(C^{2}\right)]$$

### 2.2. The Algorithm’s Description

The algorithm is described in detail as follows. Consider the set *C*. If *C* is partitioned into two convex polygons, $C^{1}$ and $C^{2}$, we observe that $C^{1}$ and $C^{2}$ do not overlap, and there must exist a straight line ℓ(a,b), defined by two points *a* and *b*, that separates $C^{1}$ and $C^{2}$. We maintain the points of *C* in two lists: P(C), the set of points defining the convex polygon, and $\bar{P}\left(C\right)$, the set of points in the interior of the convex polygon. The algorithm consists of three steps:

1. Identify the straight lines ℓ(a,b) that can separate a cluster *C*.
  Given the convexity requirements for the clusters after any partitioning, any partition of the set *C* into two convex polygons $C^{1}$ and $C^{2}$ can be achieved by introducing a straight line that crosses two points from the set P(C). Given two points $\left(x_{a},y_{a}\right)\in P\left(C\right)$ and $\left(x_{b},y_{b}\right)\in P\left(C\right)$, the line ℓ(a,b) is defined by the equation
  $$\ell \left(a,b\right)=\frac{y_{a}-y_{b}}{x_{a}-x_{b}}\left(x-x_{a}\right)+y_{a}$$
  which simplifies to
  (1) $$\ell \left(a,b\right)=\frac{y_{a}-y_{b}}{x_{a}-x_{b}}x+\frac{x_{a}\left(y_{a}-y_{b}\right)+y_{a}\left(x_{a}-x_{b}\right)}{x_{a}-x_{b}}$$
  In total, there are $\frac{\left|P(C)|\cdot |P(C)-1\right|}{2}$ such straight lines that can partition the cluster *C*.
2. Given a convex polygon *C* and a straight line ℓ(a,b), calculate the two convex polygons $C^{1}$ and $C^{2}$ separately by ℓ(a,b) using Graham’s scan algorithm [28].
  The line ℓ(a,b) partitions *C* into two sets $C^{1}$ and $C^{2}$, where $C^{1}=P\left(C^{1}\right)\cup \bar{P}\left(C^{1}\right)$ and $C^{2}=P\left(C^{2}\right)\cup \bar{P}\left(C^{2}\right)$. These sets are defined as follows:
  (2) $$\begin{array}{cc} C^{1} & =\left\{p_{i}|p_{i}\in C,\;\frac{y_{a}-y_{b}}{x_{a}-x_{b}}x_{i}+\frac{x_{a}\left(y_{a}-y_{b}\right)+y_{a}\left(x_{a}-x_{b}\right)}{x_{a}-x_{b}}\geq y_{i}\right\} \end{array}$$
  (3) $$\begin{array}{cc} C^{2} & =\left\{p_{i}|p_{i}\in C,\;\frac{y_{a}-y_{b}}{x_{a}-x_{b}}x_{i}+\frac{x_{a}\left(y_{a}-y_{b}\right)+y_{a}\left(x_{a}-x_{b}\right)}{x_{a}-x_{b}}<y_{i}\right\} \end{array}$$
  Due to the convexity of *C*, $C^{1}$, and $C^{2}$, we also have the following relationship:
  $$\begin{array}{cc} P(C) & \subseteq P\left(C^{1}\right)\cup P\left(C^{2}\right)\\ \bar{P}\left(C^{1}\right)\cup \bar{P}\left(C^{2}\right) & \subseteq \bar{P}\left(C\right) \end{array}$$
  After partitioning the set *C* using the line ℓ(a,b) into the point sets $C^{1}$ and $C^{2}$, we construct convex polygons for each set using Graham’s scan algorithm [28]. This process results in sets $P(C^{1})$, $\bar{P}\left(C^{1}\right)$, $P(C^{2})$, and $\bar{P}\left(C^{2}\right)$. Additionally, we index the points in these sets in a clockwise order for the next step.
3. Calculate the area A(C) of a convex polygon $C=P\left(C\right)\cup \bar{P}\left(C\right)$. Then, determine the maximum area that can be removed by a single partition of the cluster.
  Consider the point $\left(x_{1},y_{1}\right)\in P\left(C\right)$ and label the points clockwise on the convex polygon *C*. Using the Shoelace formula [29], the area of the convex polygon defined by the points $\left(x_{1},y_{1}\right)$, $\left(x_{2},y_{2}\right)$, *…*, $\left(x_{\left|P(C)\right|},y_{\left|P(C)\right|}\right)$ is given by
  (4) $$\begin{array}{cc} A(C) & =\sum_{i=1}^{|P(C)|-1}\left(x_{i}y_{i+1}-y_{i}x_{i+1}\right)=\frac{1}{2}|\left(\begin{array}{cc} x_{1} & x_{2}\\ y_{1} & y_{2} \end{array}\right)+\left(\begin{array}{cc} x_{2} & x_{3}\\ y_{2} & y_{3} \end{array}\right)+\cdots +\left(\begin{array}{cc} x_{\left|P(C)\right|} & x_{1}\\ y_{\left|P(C)\right|} & y_{1} \end{array}\right)| \end{array}$$
  For each line ℓ(a,b), we calculate the convex polygons resulting from the partition of the convex polygon *C*. Then, we compute the value $A\left(C\right)-[A\left(C^{1}\right)+A\left(C^{2}\right)]$. The best straight line ℓ(a,b) is chosen to minimize the maximum area removed by partitioning the set *C*.

In Algorithm 1, we present the *K*-area clustering algorithm, denoted as *K*-AC, for clustering biological data. This algorithm partitions *N* data points into at most *K* clusters, *C*, or organizes them into at most *H* hierarchical levels of clusters.
**Algorithm 1 ***K*-AC: *K*-area clustering algorithm (*N*, *C*, *K*, *H*)  1:Identify the convex hull *C* for the set of *N* data points.  2:Maintain a set *S* of convex polygons, initialized as S=C.  3:Set k=1 and h=1.  4:**while** k<K or h<H **do**  5:    define a variable δ to represent the cost reduction due to partitioning, initialized to 0;  6:    **for** each $C_{i}$ of the *k* convex polygons $C_{1},C_{2},\ldots ,C_{k}$ in *S* **do**  7:        identify the line $\ell (a_{i},b_{i})$ that partitions $C_{i}$ into two convex polygons, $C_{i}^{1}$ and $C_{i}^{2}$  8:        calculate the reduction in area, $\delta (C_{i})$, resulting from partitioning the set $C_{i}$;  9:    **end for**10:    $\delta =\max_{i}\delta \left(C_{i}\right)$;11:    $i^{*}=\arg\max_{i}\delta \left(C_{i}\right)$;12:    k←k+1;13:    update h←h+1 if the tree of $C_{1},\ldots ,C_{k}$ increases its depth by 1 due to the addition of $C_{i^{*}}^{1}$ and $C_{i^{*}}^{2}$;14:    update *S* as $S\leftarrow \left(S\setminus C_{i^{*}}\right)\cup C_{i^{*}}^{1}\cup C_{i^{*}}^{2}$;15:**end while**16:**return** *S*

Below, we present the running time complexity of *K*-AC in Theorem 1.

**Theorem** **1.** *The K-AC algorithm runs in $O(KN^{3}\log N)$ time.*

**Proof.**  Using Graham’s scan algorithm, line 1 of *K*-AC runs in O(NlogN) time. Lines 2 and 3 take constant time. The WHILE loop runs for *K* rounds. In each round, we identify $\sum_{i=1}^{k}{\left|C_{i}\right|}^{2}$ straight lines, where $\sum_{i=1}^{k}\left|C_{i}\right|=N$. For each straight line, it takes linear time $O(|C_{i}|)$ to locate $C_{i}^{1}$ and $C_{i}^{2}$ using Equations (1)–(3). The area calculations require $O(|C_{i}^{1}\left|\right)+O(|C_{i}^{2}\left|\right)=O(|C_{i}\left|\right)$ time. Identifying the best partition to reduce the total area size takes O(k) time on lines 10 and 11. Line 12 takes constant time, while Line 13 takes O(logk) time. Updating the clusters in line 14 takes $\max_{k}O(|C_{k}\left|\log\right|C_{k}\left|\right)=O\left(N\log N\right)$ time, using Graham’s scan algorithm. Thus, the total running time of the WHILE loop is

(5) $$\begin{array}{ccc} & & \sum_{k=1}^{K}\sum_{i=1}^{k}\left(O(|C_{i}{|}^{2})\left[O(|C_{i}\left|\right)+O(|C_{i}\left|\log\right|C_{i}\left|\right)+k\right]\right)\\ & \;\;\;\;= & \sum_{k=1}^{K}\sum_{i=1}^{k}\left(O(|C_{i}{|}^{2})\left[O(|C_{i}|\log|C_{i}|)+k\right]\right)\\ & \;\;\;\;\leq & \sum_{k=1}^{K}\sum_{i=1}^{k}\left(O(|C_{i}{|}^{3}\log|C_{i}|\right)+\sum_{k=1}^{K}N^{2}k\\ & \;\;\;\;\leq & O\left(\sum_{k=1}^{K}{\left(\sum_{i=1}^{k}\left|C_{i}\right|\right)}^{3}\log\sum_{i=1}^{k}\left|C_{i}\right|\right)+O\left(N^{2}K\right) \end{array}$$

(6) $$\begin{array}{ccc} & = & \sum_{k=1}^{K}O\left(N^{3}\log N)\right)+O\left(N^{2}K\right)\\ & = & O\left(KN^{3}\log N\right) \end{array}$$

Inequality (5) holds by Jensen’s inequality, and Equation (6) holds under the assumption that K≤N. □

From Theorem 1, we observe that the *K*-AC algorithm scales efficiently with the total sample size *N*, highlighting both its theoretical and practical efficiency.

## 3. Experiments

In this section, we conduct experiments to evaluate our *K*-AC algorithm. Using real biological data, we compare *K*-area clustering with other well-known clustering algorithms, including *K*-center, *K*-median, and *K*-means clustering. The experiments are conducted on a MacBook Air featuring a 2.3 GHz Dual-Core Intel Core i5 processor and 16 GB of 2133 MHz LPDDR3 memory. This MacBook Air Apple M1 was manufactured by Apple in partnership with Foxconn and Pegatron. Apple designs the processors, but Taiwan Semiconductor Manufacturing Co. (TSMC) (Hsinchu, Taiwan) builds them.

### 3.1. Datasets

To evaluate the clustering performance of the *K*-AC algorithm, we use real scRNA-seq datasets from human and mouse cells (also listed in Table 2). These datasets are downloaded from an online repository (https://hemberg-lab.github.io/scRNA.seq.datasets/, accessed on 5 January 2025) and were previously used to assess scRNA-seq clustering tools [10]. The optimal number of clusters is determined based on the true cell labels provided by the original authors’ annotations.

**Figure 2:**
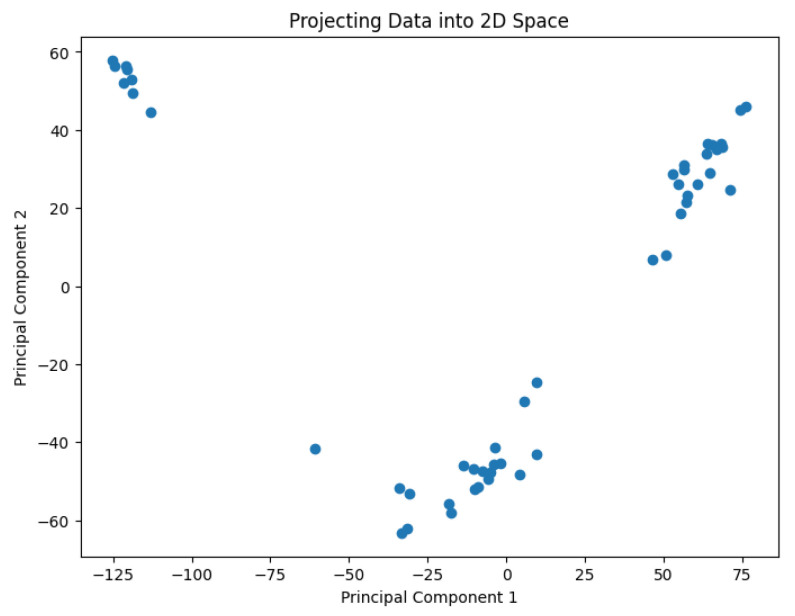
The projected data in the 2D space for the dataset Biase.

**Figure 3:**
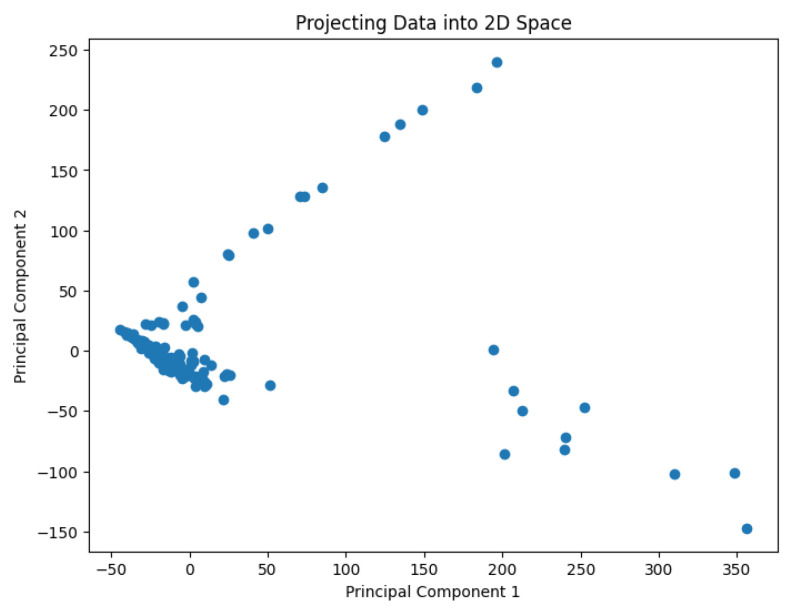
The projected data in the 2D space for the dataset Deng.

**Figure 4:**
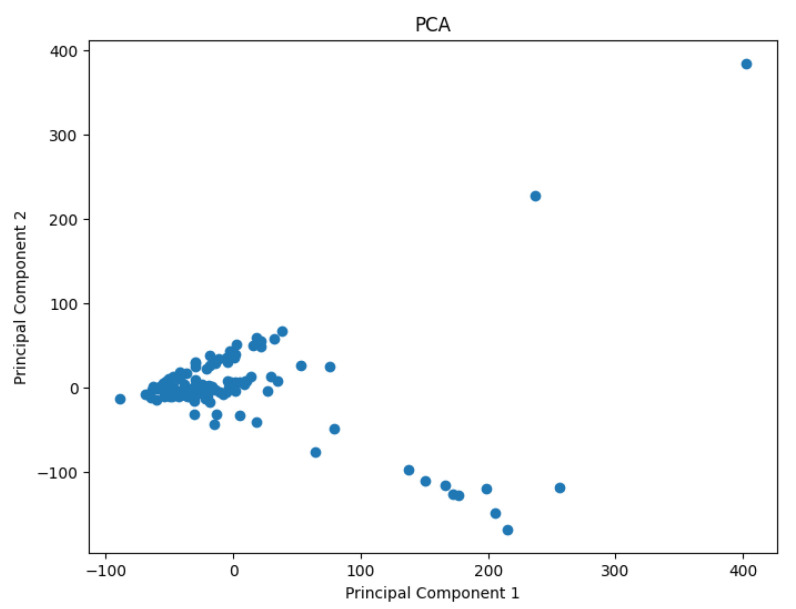
The projected data in the 2D space for the dataset Goolam.

**Figure 5:**
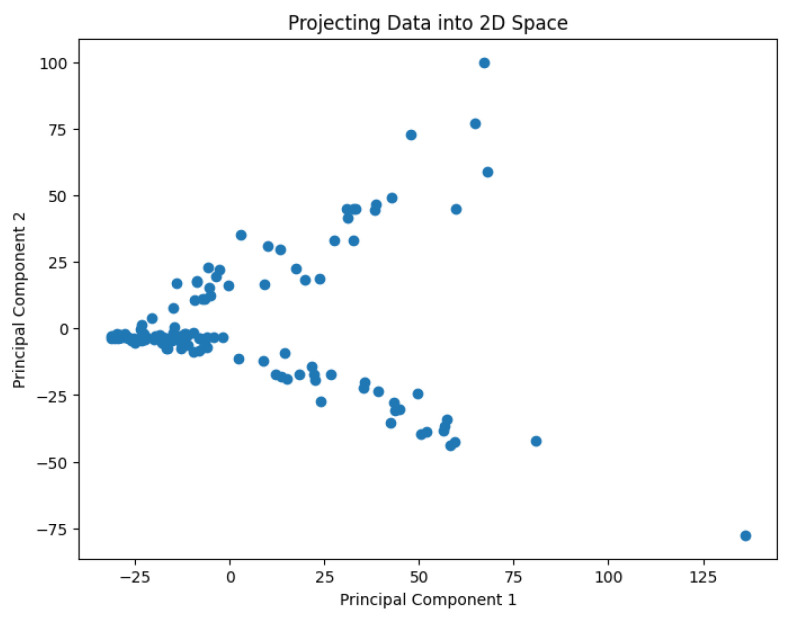
The projected data in the 2D space for the dataset Ting.

**Figure 6:**
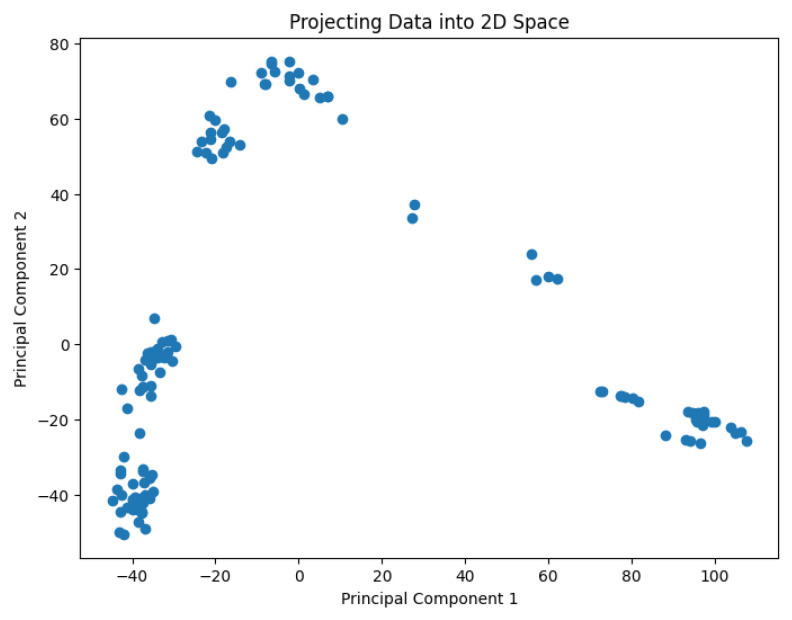
The projected data in the 2D space for the dataset Yan.

A pre-processing step involves applying the PCA analysis to reduce the dimensionality of the data and generate values representing them in a 2D space. The use of PCA to highlight information relevant to the classification of genes or cell lines has been studied by Crescenzi and Giuliani [26] in the context of microarray analysis. Their work uncovers mutual similarities from cluster analysis among differently labeled statistical units, generating new classifications at varying levels of detail that can be interpreted in biological terms. The PCA results are shown in Figure 2, Figure 3, Figure 4, Figure 5 and Figure 6.

### 3.2. Metrics

We set K=3,10,5,7,7 for the Biase, Deng, Goolam, Ting, and Yan datasets, respectively. We then measure the cost of the following clustering algorithms: the *K*-center clustering algorithm [29], the *K*-median clustering algorithm [30], the *K*-means Lloyd’s clustering algorithm [21] and our proposed *K*-area clustering algorithm *K*-AC. To evaluate the performance of these clustering algorithms, we use Normalized Mutual Information (NMI) [31] to assess the quality of the generated clusters.

NMI is a widely used metric for evaluating clustering performance by measuring the mutual dependence between the true labels and the predicted clusters. The mutual information is normalized to ensure values between 0 and 1, where 1 indicates perfect clustering and 0 implies no mutual information between the clustering and ground truth. The NMI between two cluster assignments *C* (ground truth) and $C^{\prime }$ (predicted clusters) is computed as

$$\begin{array}{c} NMI\left(C,C^{\prime }\right)=\frac{I(C,C^{\prime })}{\sqrt{En\left(C\right)En\left(C^{\prime }\right)}} \end{array}$$

where $I(C,C^{\prime })$ is the mutual information between *C* and $C^{\prime }$, and En(C) and $En(C^{\prime })$ are the entropy of *C* and $C^{\prime }$, respectively.

### 3.3. K-Center Clustering Algorithm

The *K*-center clustering algorithm aims to find a partition $C=\{C_{1},C_{2},\ldots ,C_{K}\}$ of the data points into *K* clusters, with corresponding centers $c_{1},c_{2},\ldots ,c_{K}$ such that the maximum distance between any data point and the center of its assigned cluster is minimized. Specifically, the goal is to minimize

$$\max_{j=1}^{K}\max_{p_{i}\in C_{j}}d\left(p_{i},c_{j}\right)$$

**Figure 7:**
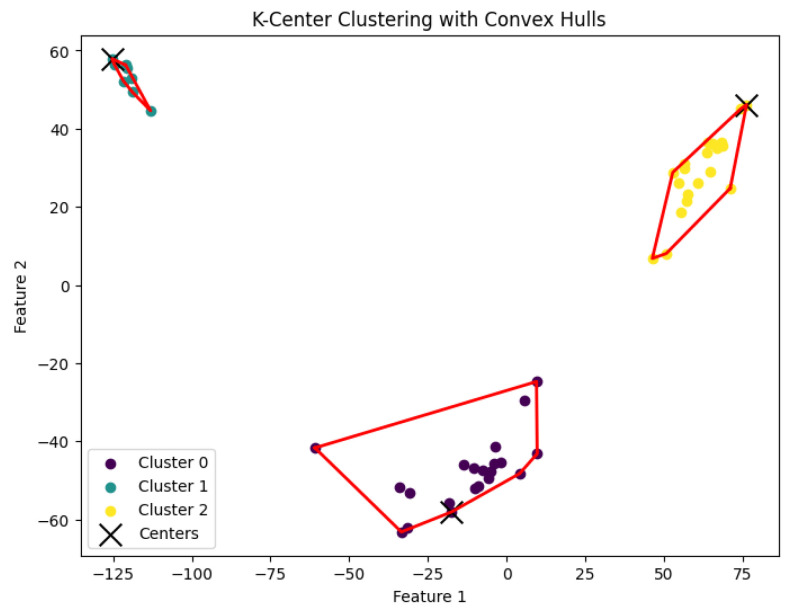
The *K*-center clustering algorithm’s result with K=3 for the dataset Biase.

**Figure 8:**
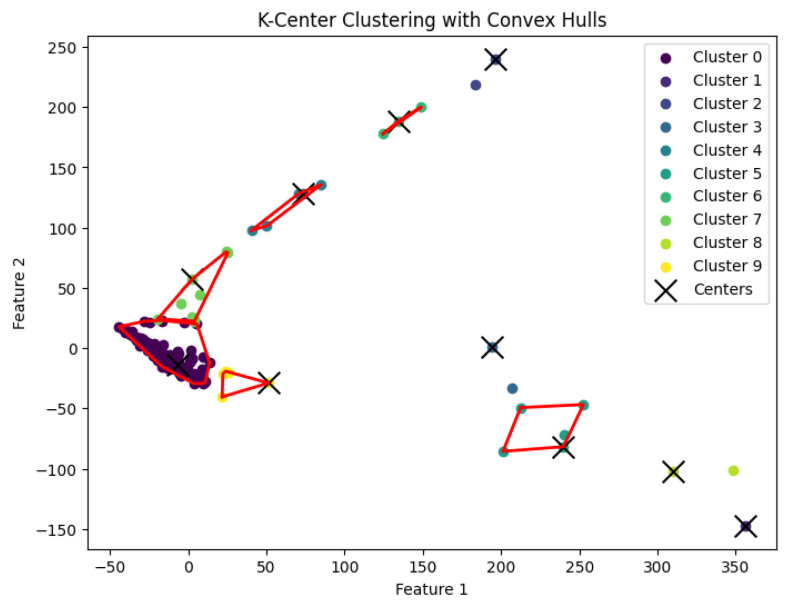
The *K*-center clustering algorithm’s result with K=10 for the dataset Deng.

**Figure 9:**
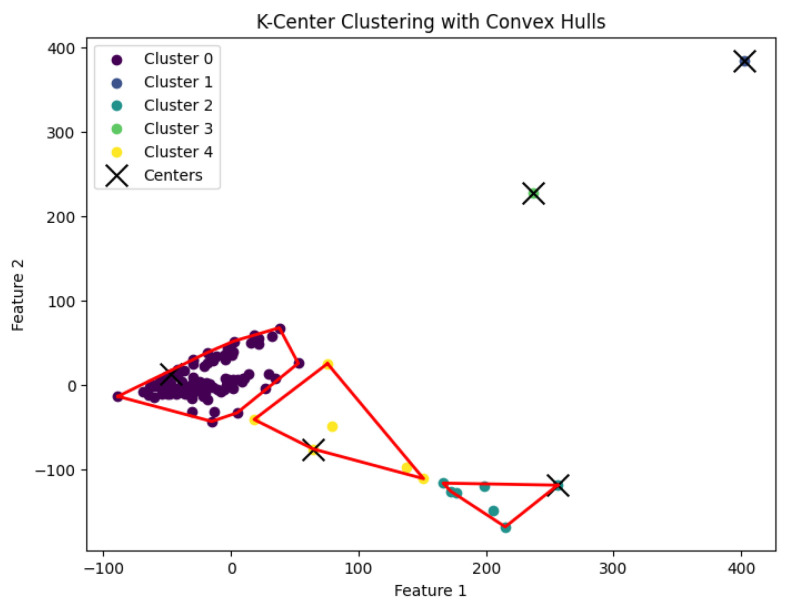
The *K*-center clustering algorithm’s result with K=5 for the dataset Goolam.

**Figure 10:**
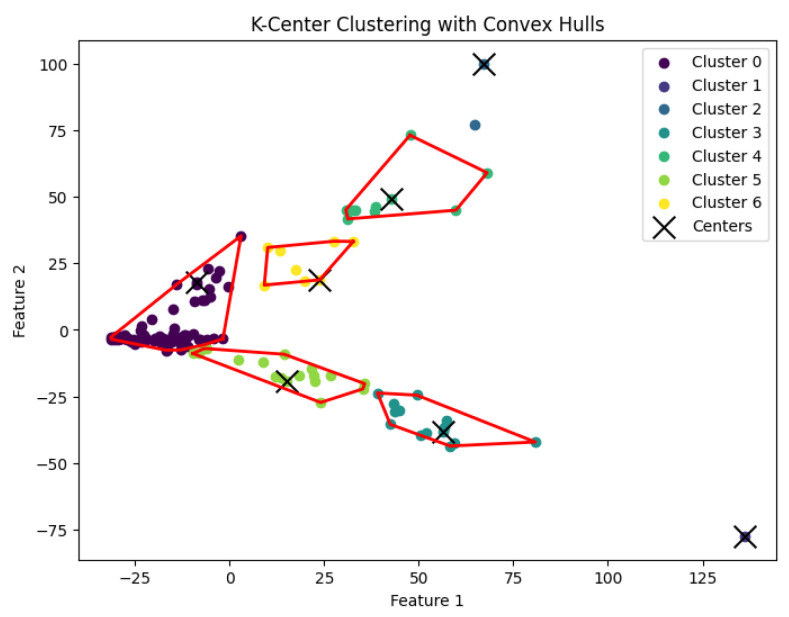
The *K*-center clustering algorithm’s result with K=7 for the dataset Ting.

**Figure 11:**
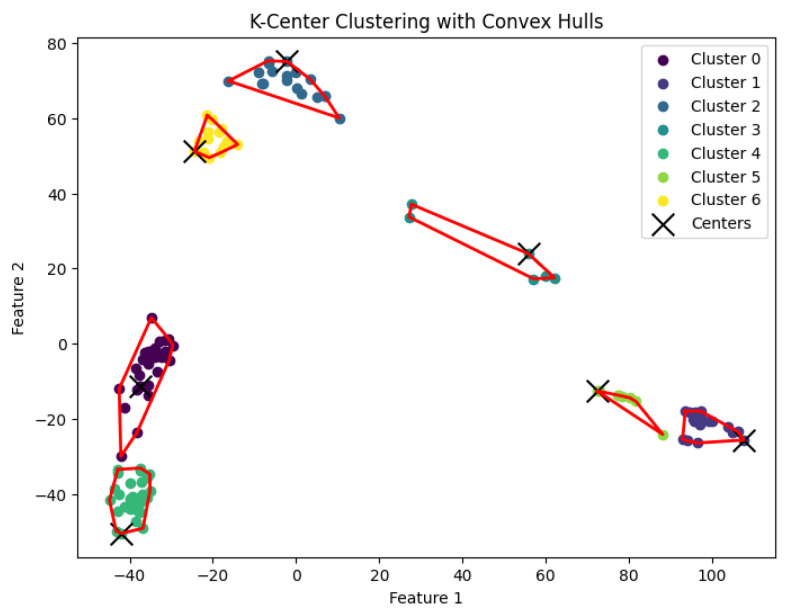
The *K*-center clustering algorithm’s result with K=7 for the dataset Yan.

When *K* is not a fixed input or may vary as a function of the number of data points *N*, this *K*-center clustering problem becomes NP-hard. In our experiments, we apply the farthest-traversal algorithm to solve the *K*-center problem and generate clusters. The resulting clustering is shown in Figure 7, Figure 8, Figure 9, Figure 10 and Figure 11.

### 3.4. K-Median Clustering Algorithm

The *K*-median clustering algorithm seeks to find a partition $C=\{C_{1},C_{2},\ldots ,C_{K}\}$ of the data points into *K* clusters, with corresponding centers $c_{1},c_{2},\ldots ,c_{K}$ to minimize the total distance between each data point and the center of its assigned cluster. Specifically, the objective is to minimize

$$\sum_{j=1}^{K}\sum_{p_{i}\in C_{j}}d\left(p_{i},c_{j}\right)$$

**Figure 12:**
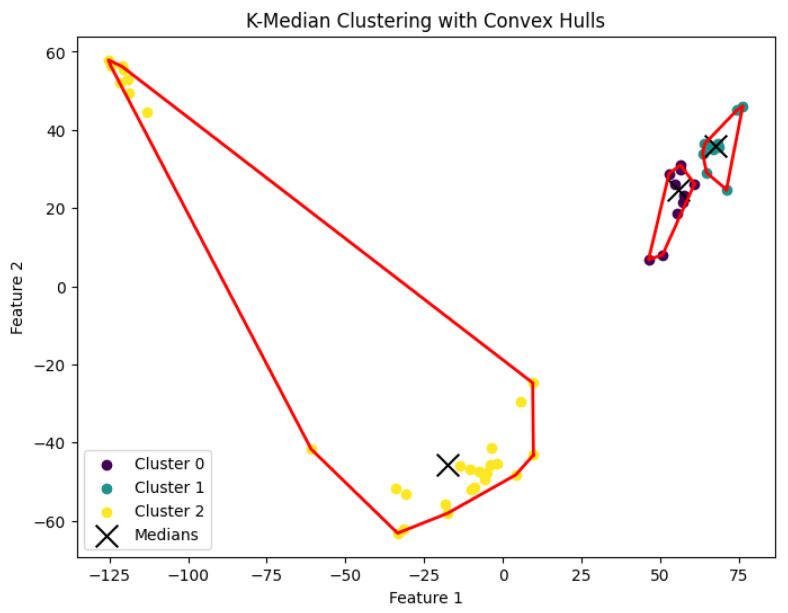
The *K*-median clustering algorithm’s result with K=3 for the dataset Biase.

**Figure 13:**
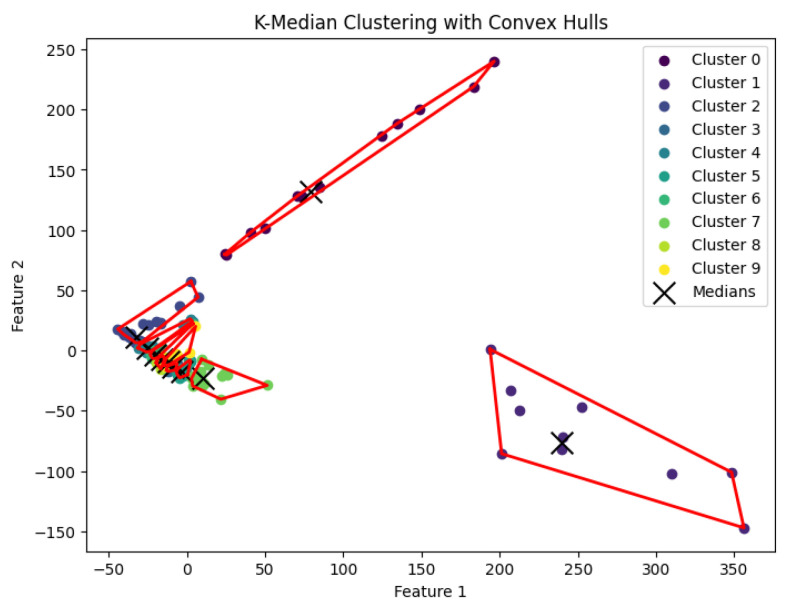
The *K*-median clustering algorithm’s result with K=10 for the dataset Deng.

**Figure 14:**
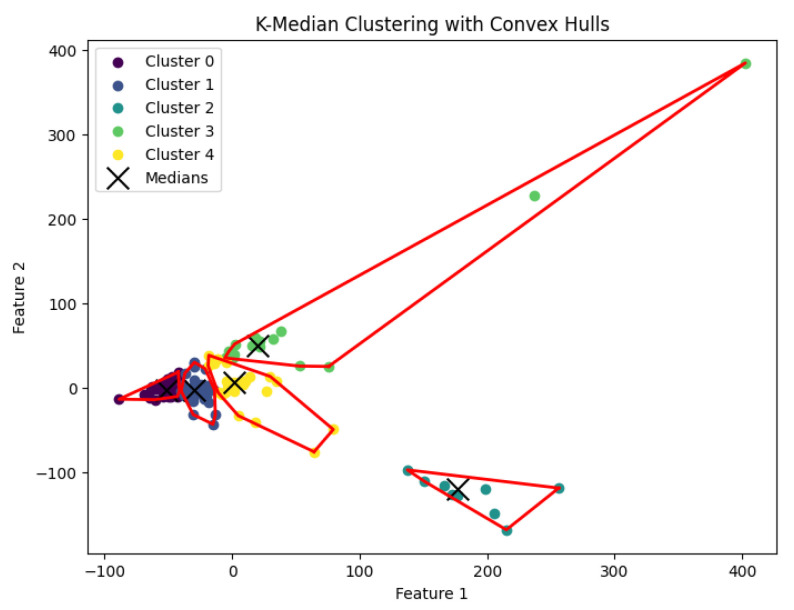
The *K*-median clustering algorithm’s result with K=5 for the dataset Goolam.

**Figure 15:**
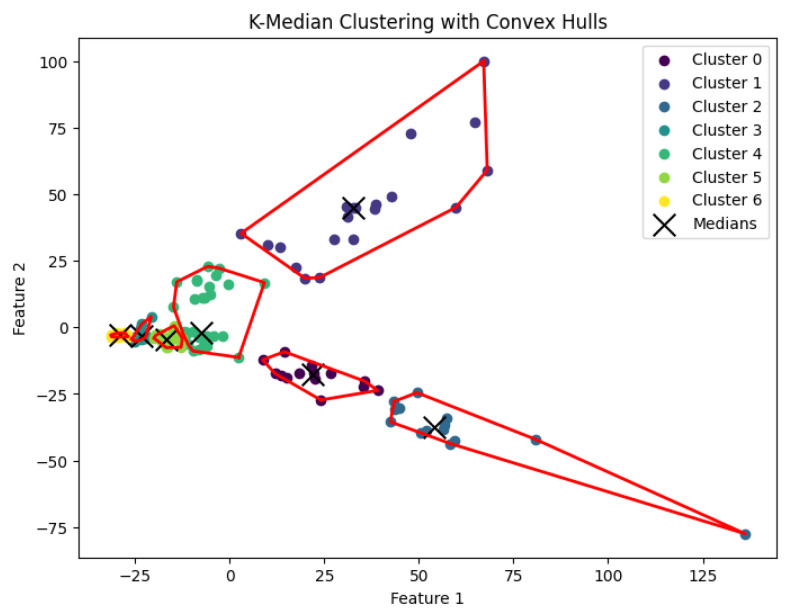
The *K*-median clustering algorithm’s result with K=7 for the dataset Ting.

**Figure 16:**
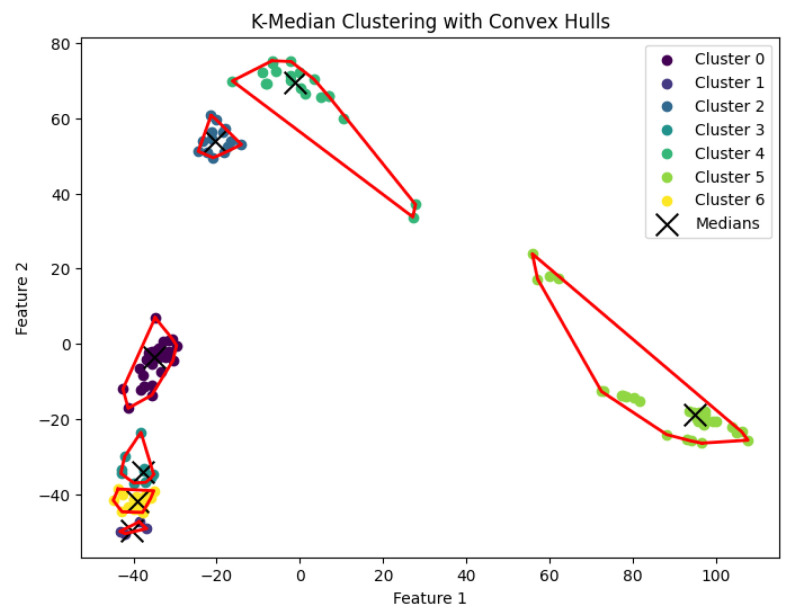
The *K*-median clustering algorithm’s result with K=7 for the dataset Yan.

When *K* is not part of an input or may be a function of *n*, then this *K*-median clustering problem is NP-hard. In our experiments, we run the Lloyd-style iteration algorithm [32] for the *K*-median clustering problem to generate clusters. The resulting clustering is shown in Figure 12, Figure 13, Figure 14, Figure 15 and Figure 16.

### 3.5. K-Means Clustering Algorithm

The *K*-means clustering algorithm aims to find a partition $C=\{C_{1},C_{2},\ldots ,C_{K}\}$ of the data points into *K* clusters, with corresponding centers $c_{1},c_{2},\ldots ,c_{K}$ such that the sum of squared distances between each data point and the center of its assigned cluster is minimized. Specifically, the objective is to minimize

$$\sum_{j=1}^{K}\sum_{p_{i}\in C_{j}}d^{2}\left(p_{i},c_{j}\right)$$

**Figure 17:**
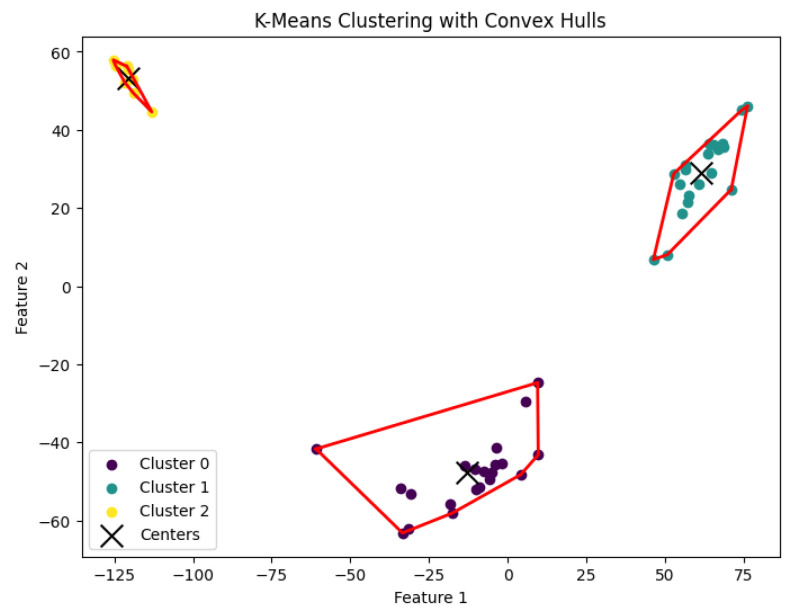
The *K*-means clustering algorithm’s result with K=3 for the dataset Biase.

**Figure 18:**
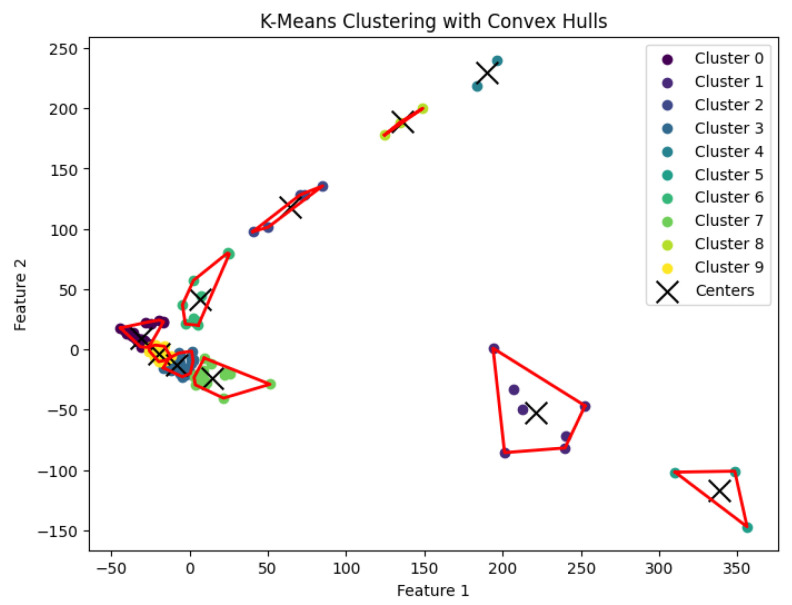
The *K*-means clustering algorithm’s result with K=10 for the dataset Deng.

**Figure 19:**
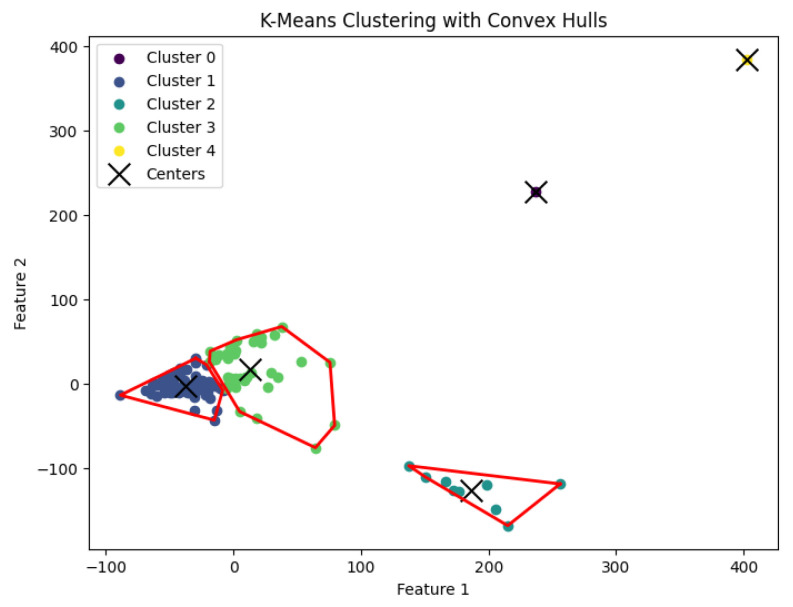
The *K*-means clustering algorithm’s result with K=5 for the dataset Goolam.

**Figure 20:**
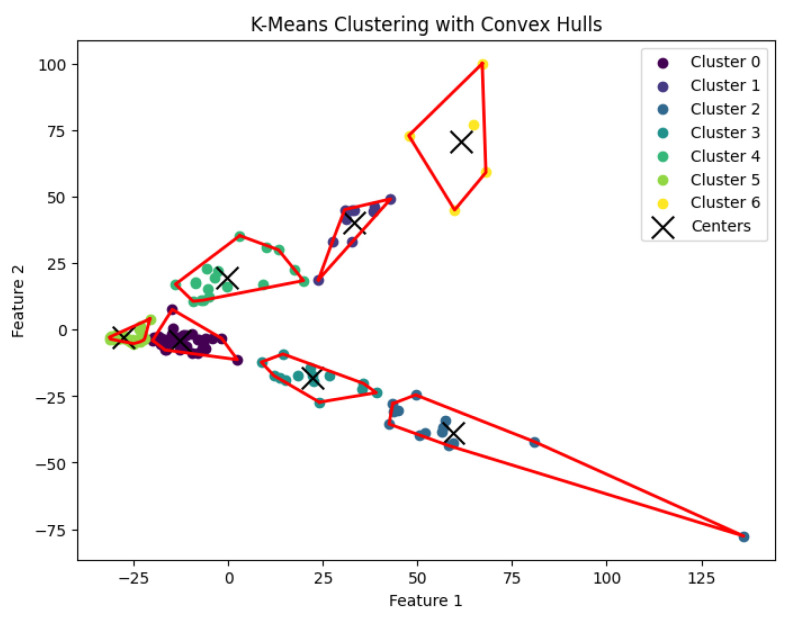
The *K*-means clustering algorithm’s result with K=7 for the dataset Ting.

**Figure 21:**
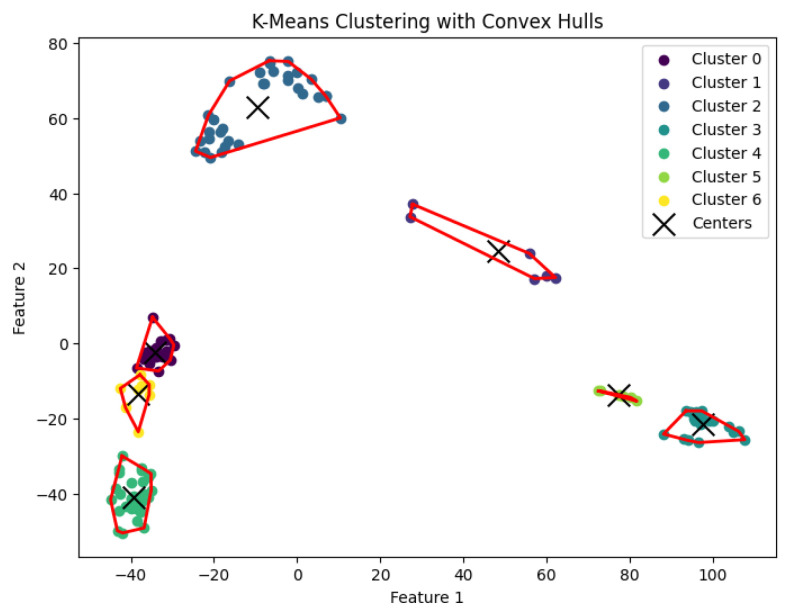
The *K*-means clustering algorithm’s result with K=7 for the dataset Yan.

When *K* is not part of an input or may be a function of *n*, then this *K*-means clustering problem is NP-hard. In our experiments, we run Llyod’s algorithm [21] for the *K*-means clustering problem to generate clusters. The resulting clustering is shown in Figure 17, Figure 18, Figure 19, Figure 20 and Figure 21.

### 3.6. K-Area Clustering Algorithm

The *K*-area clustering algorithm aims to find a partition $C=\{C_{1},C_{2},\ldots ,C_{K}\}$ of the data points into *K* clusters such that the sum of convex hull areas is minimized. Specifically, the objective is to minimize

$$\sum_{j=1}^{K}A\left(C_{j}\right)$$

where $A(C_{j})$ denotes the area of the region defined by the polygon $C_{j}$.

**Figure 22:**
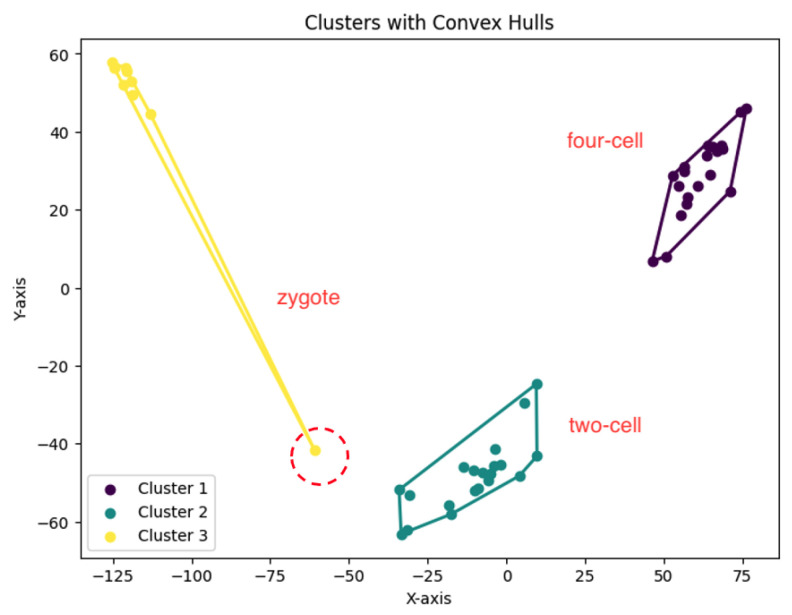
The *K*-area clustering algorithm’s result with K=3 for the dataset Biase. The red dotted circle highlights an outlier.

**Figure 23:**
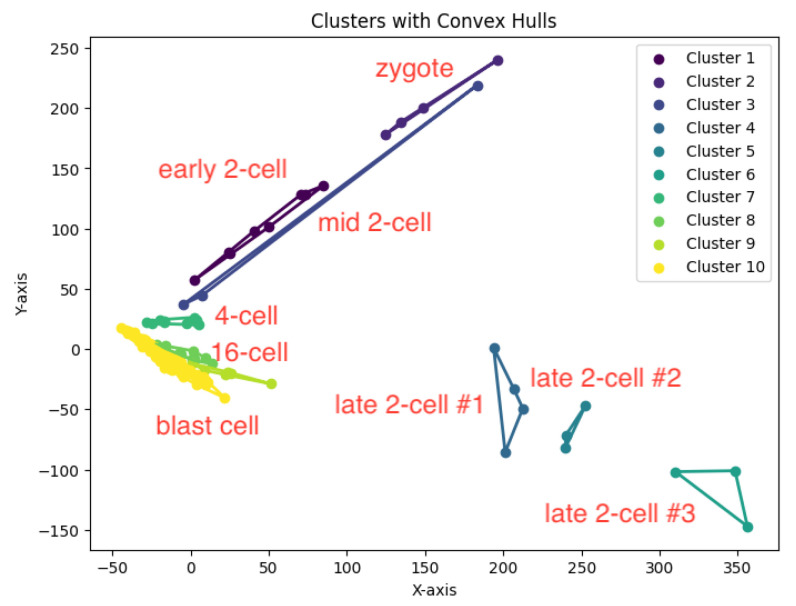
The *K*-area clustering algorithm’s result with K=10 for the dataset Deng.

**Figure 24:**
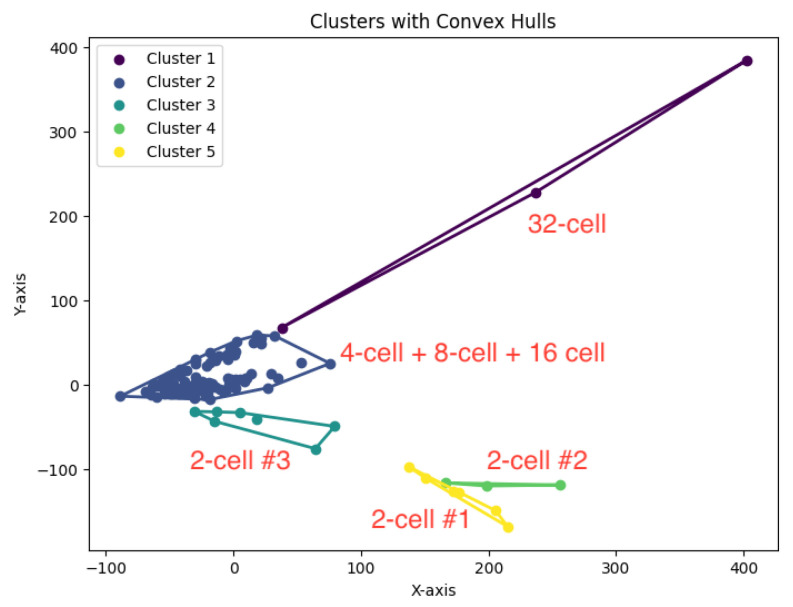
The *K*-area clustering algorithm’s result with K=5 for the dataset Goolam.

**Figure 25:**
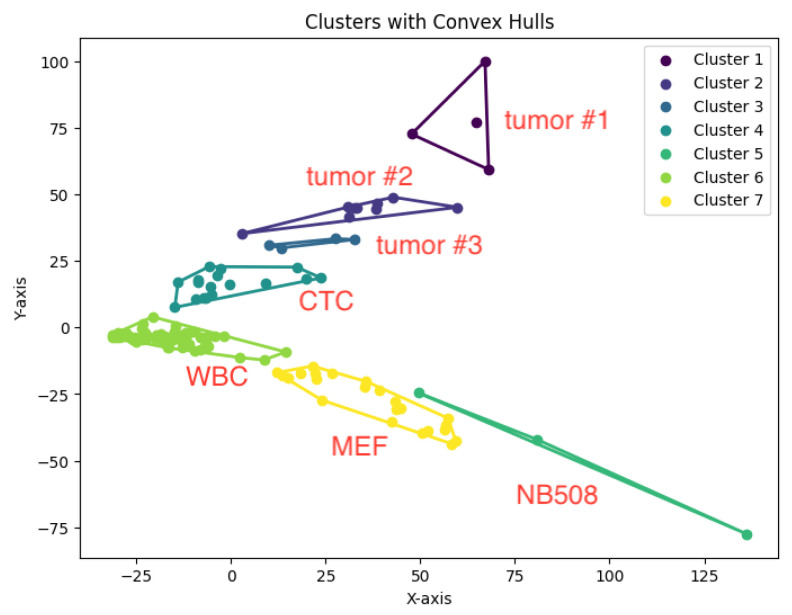
The *K*-area clustering algorithm’s result with K=7 for the dataset Ting. MEF: mouse embryonic fibroblast; WBC: white blood cell; NB508: pancreatic cancer cell line; CTC: circulating tumor cell.

**Figure 26:**
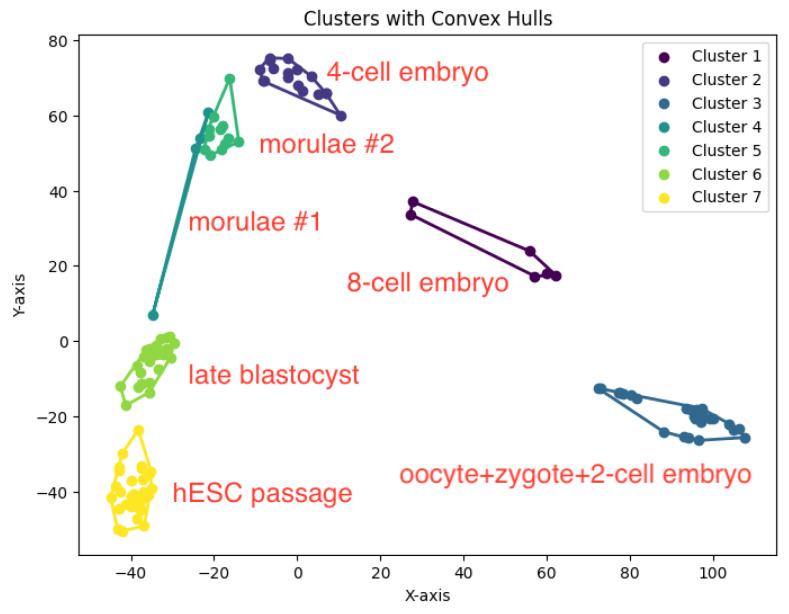
The *K*-area clustering algorithm’s result with K=7 for the dataset Yan.

In our experiments, we run our algorithm *K*-AC for the *K*-area clustering problem to generate clusters. The resulting clustering is shown in Figure 22, Figure 23, Figure 24, Figure 25 and Figure 26.

### 3.7. Performance Comparison

We summarize the NMI scores for these algorithms in Table 3. Our algorithm, *K*-AC, outperforms all other algorithms in three cases but is slightly less effective in two: the *K*-median algorithm on the Deng dataset and on the Ting dataset. We also evaluate the results from a biological perspective by utilizing the experimental labels provided by the original authors’ analysis. Across all datasets, the *K*-AC algorithm demonstrates a remarkable ability to accurately group outlier cells, which exhibits significant distances from the majority of cells within the same cell type. For example, in the Biase dataset [33], *K*-AC correctly clusters one outlier cell (a zygotic cell highlighted in the red dotted circle in Figure 22), while the other three methods incorrectly group it with its neighboring cluster (see Figure 7, Figure 12 and Figure 17). By identifying these outlier cells, the *K*-AC algorithm provides valuable insights into cellular heterogeneity, potentially leading to novel biological discoveries. These results underscore that *K*-AC offers a biologically relevant and geometrically interpretable clustering criterion, enabling more meaningful clusterings of high-dimensional biological data compared to the other three algorithms.

**Figure 27:**
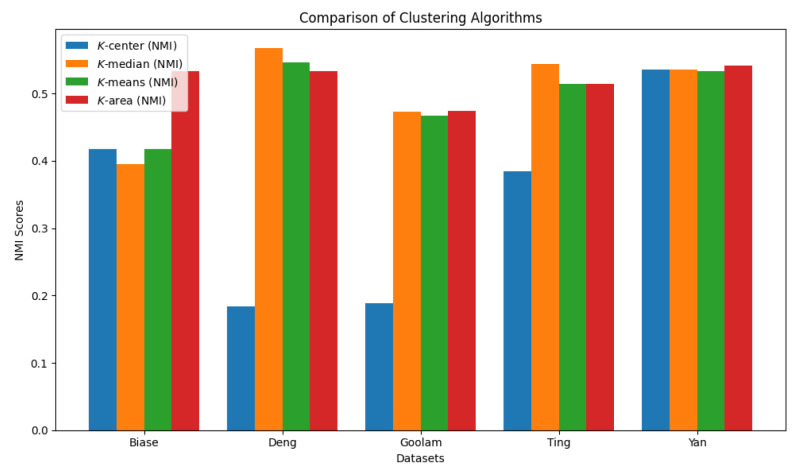
Comparison of four clustering algorithms based on their performance, evaluated using NMI values.

**Figure 28:**
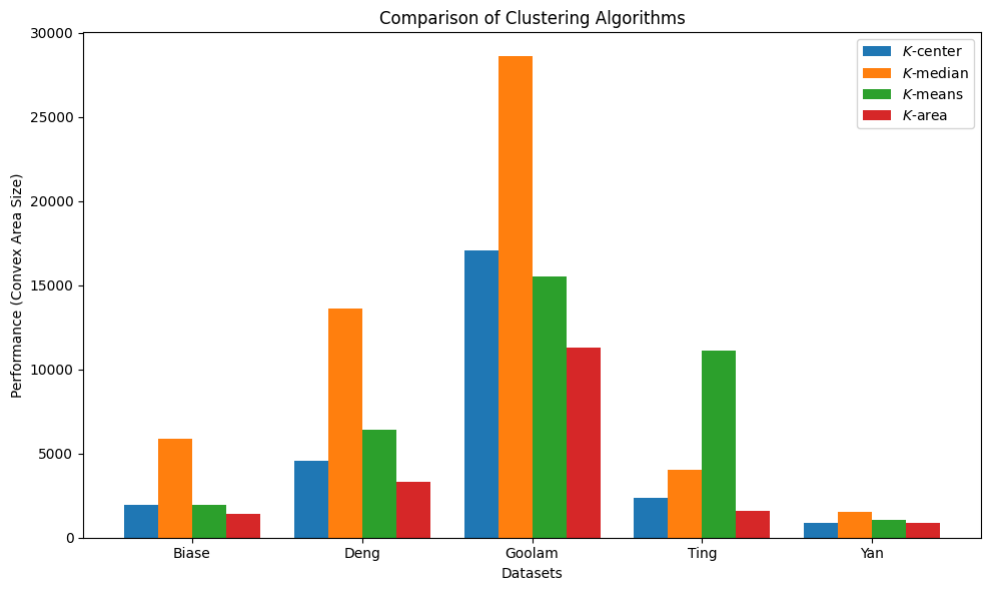
Comparison of four clustering algorithms based on their performance, evaluated using convex area sizes.

We report the algorithms’ running time and the convex areas produced by these four clustering algorithms in Table 4 and Table 5, respectively. For some instances, the *K*-AC algorithm runs faster than the other clustering algorithms; however, when the value of *K* is large, the *K*-AC algorithm runs slower. It is evident that the *K* area algorithm results in the smallest convex area, and in some cases, such as the Goolam dataset, it reduces the space by up to 60%. The comparison results are visualized in Figure 27 and Figure 28.

### 3.8. Hierarchical Clustering

To demonstrate the effectiveness of the *K*-area algorithm in interpreting the hierarchical structure of biological data—particularly in identifying the number of natural clusters—we conduct experiments to explore the construction of the cluster tree. We use the ratio of the convex hull areas between two neighboring rounds as a criterion for determining when to halt the partitioning of existing clusters.

**Figure 29:**
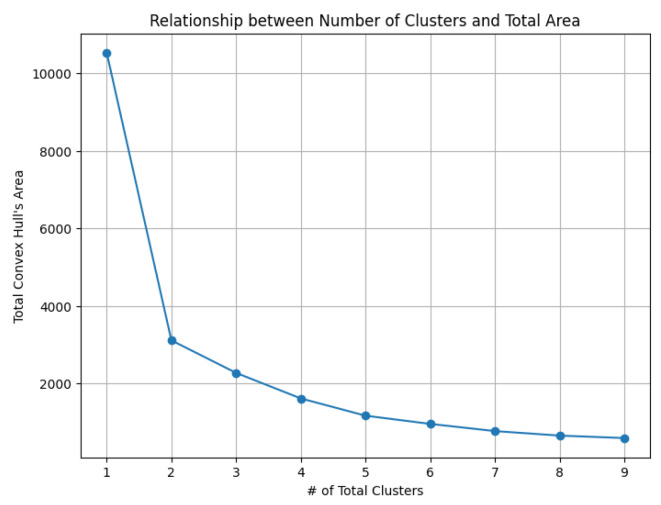
The area ratios reveal the number of clusters needed.

The dataset Yan used in our experiments has an optimal number of clusters, $K^{*}=7$, as provided by the authors’ original annotation [34]. We calculate the total cluster areas for k=1,2,…,9 and summarize the results in Table 6, with visualizations in Figure 29. The area ratio between two neighboring rounds increases from 0 to 1. As the ratio approaches 1, the benefit of further partitioning into additional clusters diminishes. Notably, after k=7, the area ratio stabilizes near 1, indicating that further partitioning yields diminishing returns. This feature of the *K*-area algorithm aids in identifying an appropriate value for *K*, the number of clusters.

**Figure 30:**
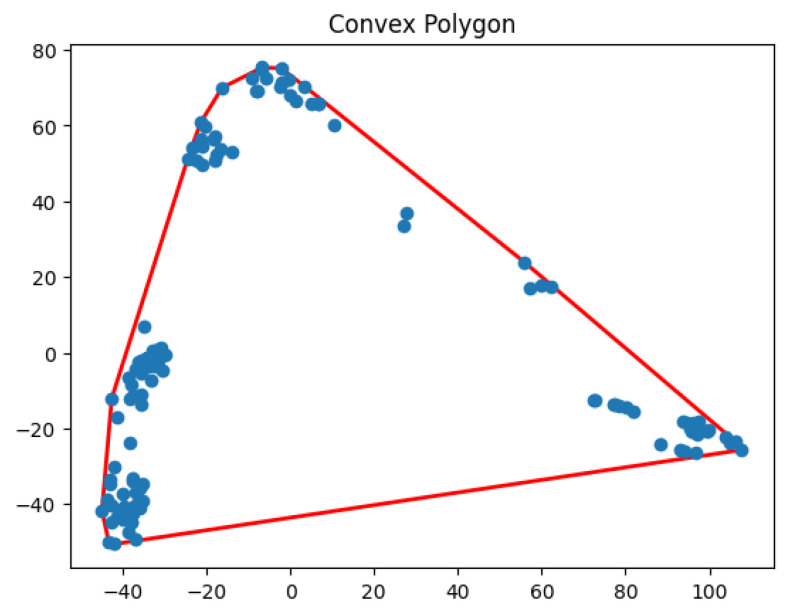
One cluster for Yan.

**Figure 31:**
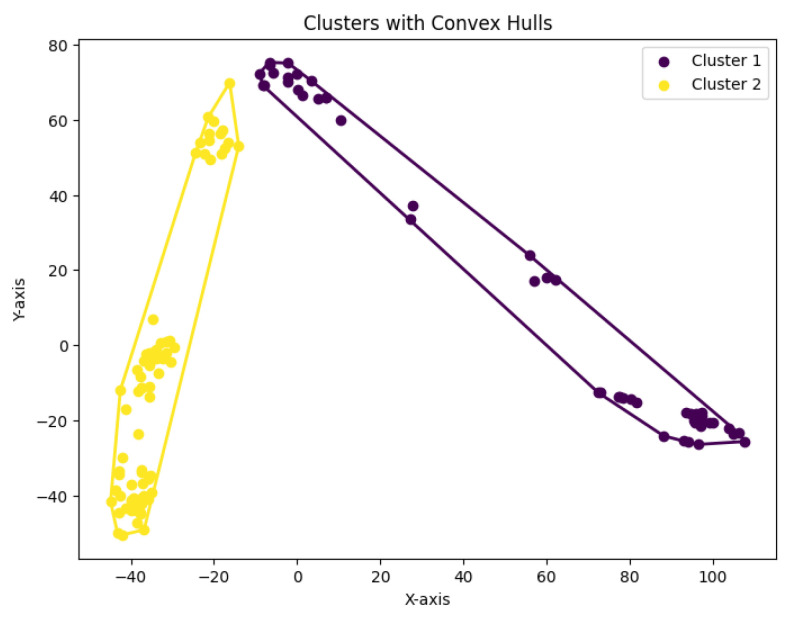
Two clusters for Yan.

**Figure 32:**
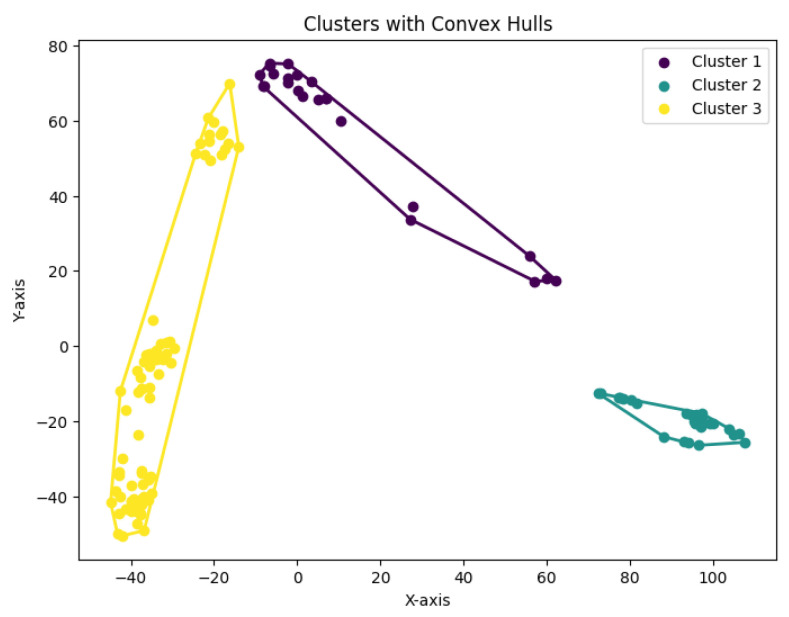
Three clusters for Yan.

**Figure 33:**
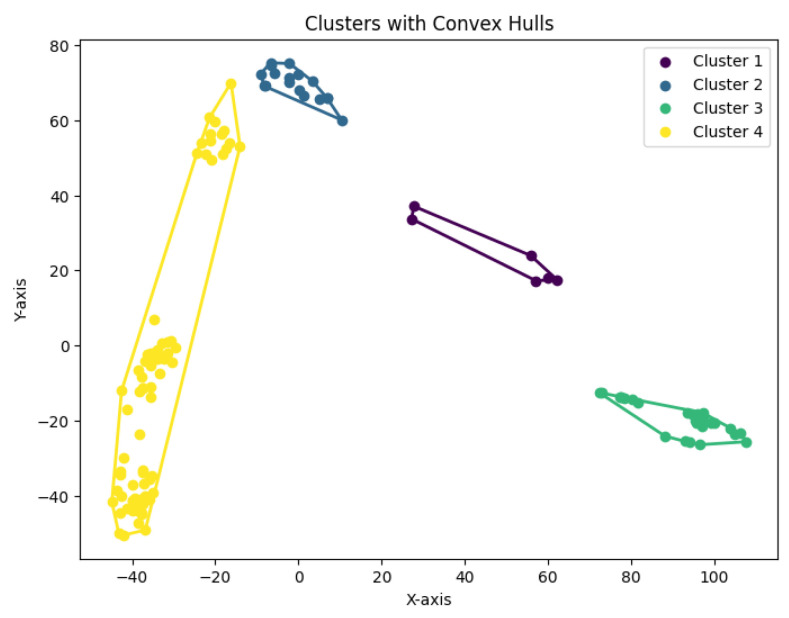
Four clusters for Yan.

**Figure 34:**
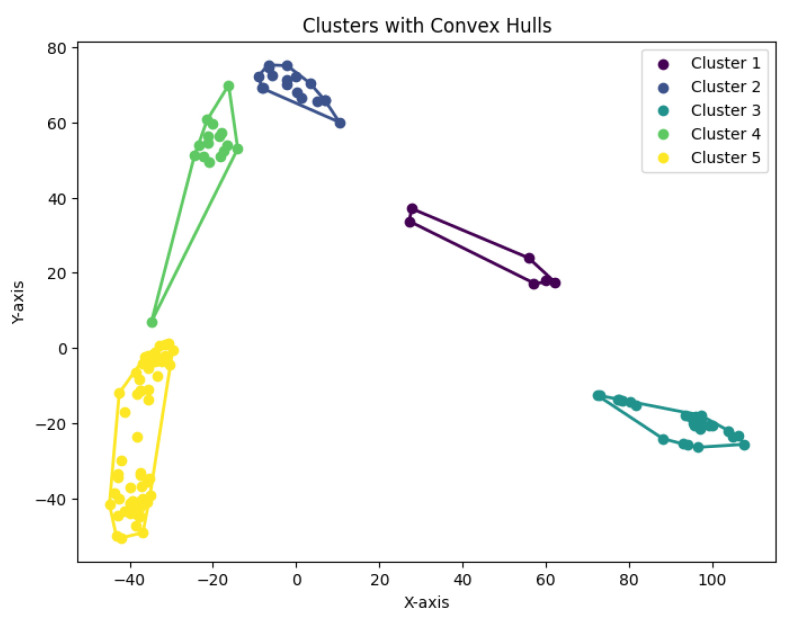
Five clusters for Yan.

**Figure 35:**
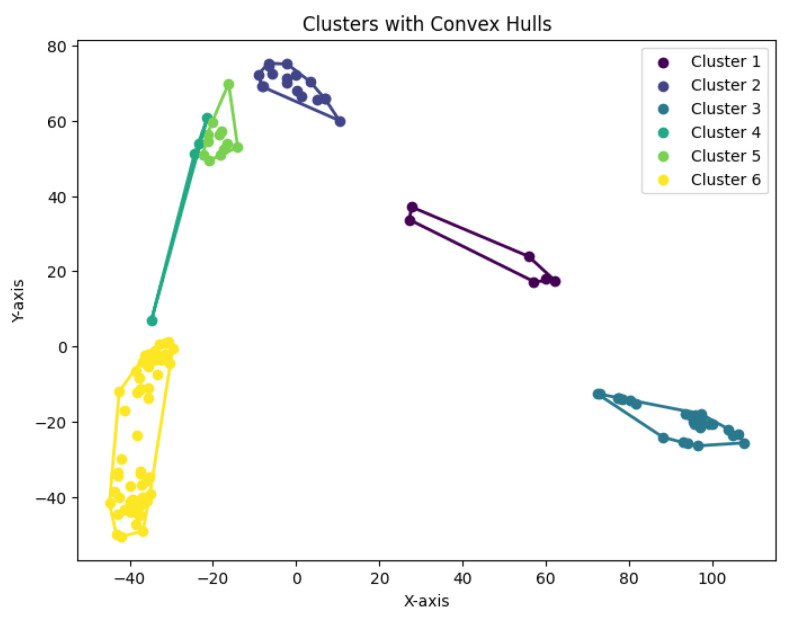
Six clusters for Yan.

**Figure 36:**
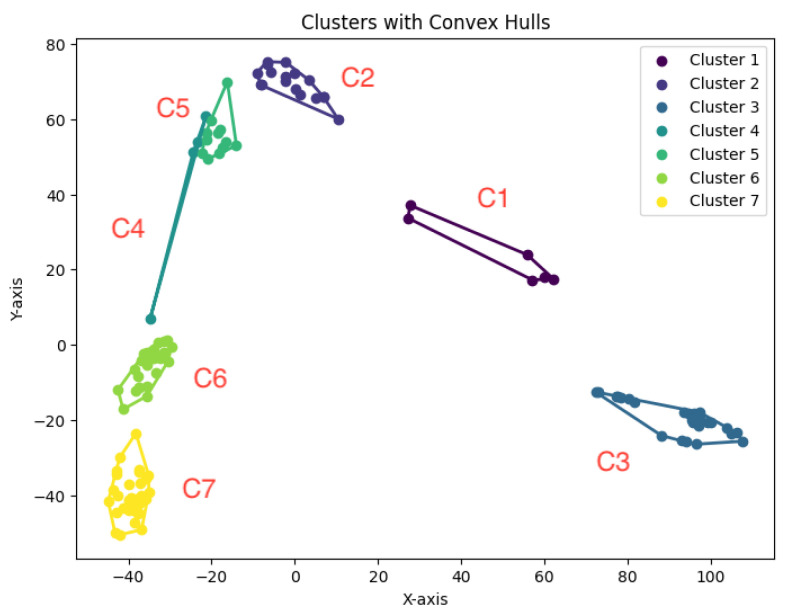
Seven clusters for Yan.

**Figure 37:**
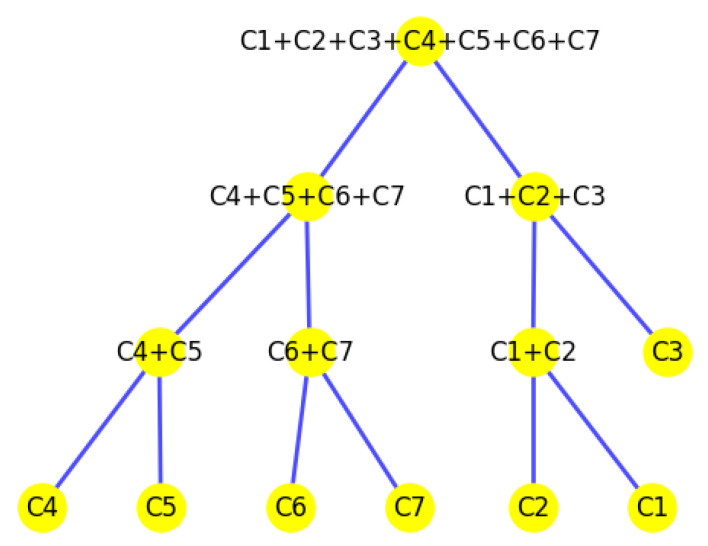
The tree structure representing the partition made by the *K*-area algorithm.

We construct the cluster tree generated by the *K*-area algorithm to visually represent its progression for the dataset Yan from Figure 30, Figure 31, Figure 32, Figure 33, Figure 34, Figure 35 and Figure 36. The tree structure of the partitions is shown in Figure 37. In this tree, the root node represents the initial convex hull, with each parent node (a cluster) being divided into two child nodes (subclusters). The leaves of the tree correspond to the final set of clusters. The height of the tree is constrained by the given hierarchical level *H*. By examining the authors’ labels for the cell types [34], we observe that the hierarchical tree organization aligns well with biological relationships. Specifically, C1 (4-cell embryo) is closely related to C2 (8-cell embryo), and they are initially merged, reflecting their sequential developmental stages. The combined group of C1 and C2 then merges with C3, which consists of a heterogeneous mixture of Oocyte, Zygote, and 2-cell embryo cells, indicating their shared early embryonic origin. C4 and C5 represent two distinct groups of Morulae cells, maintaining their biological distinction. Additionally, C6 (Late blastocyst) and C7 (hESC passage) cluster together, consistent with their advanced differentiation stages. This biologically meaningful clustering validates the hierarchical structure inferred by the *K*-AC algorithm.

## 4. Summary and Discussion

A key innovation of the *K*-volume algorithm is its ability to simultaneously optimize both the hierarchical structure and the number of clusters at each level, leveraging nonlinear optimization. Unlike traditional clustering methods, which require a predefined number of clusters, our method dynamically determines the optimal number of clusters and hierarchical layers based on the convex volume distribution. This adaptive nature makes it particularly well suited for complex biological datasets, such as scRNA-seq and spatial transcriptomics, where cell populations exhibit multi-scale hierarchical relationships.

Moreover, the flexibility of our method extends beyond biological applications, making it a powerful tool for a wide range of data-intensive domains, including finance, social network analysis and image segmentation, where hierarchical patterns naturally emerge. The method holds significant potential as a core component in modern data analysis pipelines. By integrating this method into widely used computational frameworks, we envision it becoming a standard clustering technique for high-dimensional and hierarchical data, offering a versatile and scalable solution for data-driven discovery.

Looking ahead, we will investigate incorporating performance metrics into the clustering of datasets. This consideration arises from the observation that the *K*-area algorithm performs slightly worse than the *K*-median algorithm on two datasets (Table 1). We believe this is due to the metric used for degenerate polygons. We define the area of a degenerate polygon as 0, which may result in multiple lines with zero area, where the line endpoints are close to each other. In such cases, we may consider incorporating the *K*-median algorithm’s metric. The optimal combination of performance metrics for various data points is currently under investigation.

Our future research will focus on extending this framework by incorporating advanced high-dimensional data processing techniques, such as dimensionality reduction methods and deep learning-based feature extraction, to further enhance clustering performance [35,36]. We also plan to integrate multi-omics data, including single-cell spatial transcriptomics [37], epigenomics [38], and proteomics [39], to create a more holistic representation of cellular states and regulatory mechanisms. By combining these advancements, we aim to develop a powerful, scalable clustering framework capable of uncovering complex biological patterns across multiple layers of molecular information, ultimately contributing to a deeper understanding of cellular functions and disease mechanisms. Note that in [40], it was reported that cultured cells exhibit high correlations between the values of certain shape descriptors, with the geometrical features postulated to be responsible for each correlation. Additionally, these correlations show a complex dependence on the size, shape, and number of invaginations. We plan to further investigate the geometrical characteristics of various cells and provide more biological interpretations for the clusters generated based on our geometrical approach.

## 5. Conclusions

In this paper, we address the challenge of clustering biological data by developing a novel algorithm based on the total convex volume encompassed by points within the same cluster. This approach offers a biologically relevant and geometrically interpretable clustering criterion, enabling more meaningful groupings of high-dimensional biological data. We introduce the *K*-volume algorithm, which effectively balances accuracy and computational feasibility. Experimental evaluations demonstrate the algorithm’s effectiveness compared to well-established clustering methods, showing promising results in capturing biological structures and relationships within the data.

## Figures and Tables

**Table 1:** Different types of clustering algorithms. ∗: denotes our approach in this paper.

Center-Based Clusters			High-Density Clusters	Center-Based Density Clusters ^∗^
*K*-center clustering	*K*-median clustering	*K*-means clustering	*K*-high-density regions	*K*-volume clustering ^∗^
NP-hard	NP-hard	NP-hard	NP-hard	-
An iterative greedy algorithm [4]	An iterative greedy algorithm [5]	An iterative greedy algorithm [24]	An iterative greedy algorithm [25]	An iterative greedy algorithm ^∗^

**Table 2:** Datasets that we use for the experimental study of clustering algorithms.

Name	Size (# of Cells)	Size (# of Genes)	$K^{*}$: # of Optimal Clusters	PubMed ID
Biase	49	25,738	3	25096407
Deng	268	22,431	10	24408435
Goolam	124	41,480	5	27015307
Ting	149	29,018	7	25242334
Yan	90	20,214	7	23934149

**Table 3:** Detailed NMI scores of four clustering algorithms. We use ^∗^ to denote the best results.

Dataset/Algorithms	*K*-Center	*K*-Median	*K*-Means	*K*-Area
Biase	0.417	0.395	0.417	0.533 ^∗^
Deng	0.184	0.567 ^∗^	0.546	0.533
Goolam	0.188	0.473	0.467	0.474 ^∗^
Ting	0.384	0.544 ^∗^	0.514	0.514
Yan	0.535	0.536	0.533	0.542 ^∗^

**Table 4:** Comparison of four clustering algorithms’ running time (in seconds).

Dataset/Algorithms	*K*-Center	*K*-Median	*K*-Means	*K*-Area
Biase	23	20	21	5
Deng	26	25	28	641
Goolam	20	23	22	59
Ting	19	20	22	66
Yan	14	14	19	50

**Table 5:** Detailed convex area sizes of four clustering algorithms.

Dataset/Algorithms	*K*-Center	*K*-Median	*K*-Means	*K*-Area
Biase	1946.17	5872.96	1946.21	1390.24
Deng	4557.64	13,640.09	6390.81	3278.49
Goolam	17,083.75	28,588.06	15,519.73	11,270.08
Ting	2376.34	4016.70	11,114.86	1588.39
Yan	857.88	1516.40	1053.21	854.69

**Table 6:** The total clusters’ area and the ratios of the convex hull areas between two neighboring rounds help determine the value *K*.

# of Total Cluster	Total Convex Hull’s Area	Area Ratios of Two Neighboring Rounds
1	10,530.41	0
2	3110.62	0.30
3	2271.75	0.73
4	1613.22	0.71
5	1167.92	0.72
6	956.11	0.82
7	770.70	0.81
8	654.54	0.85
9	590.55	0.90

## Data Availability

The research data are stored at https://drive.google.com/drive/folders/1OJdP3UjZKXrvFx4QsIyhG1-l7LX2Tikc?usp=sharing (accessed on 2 February 2025). The code is available at GitHub https://github.com/aqcc-va/clustering (accessed on 3 February 2025).
